# Gold Nanoparticles Deposited on Surface Modified Carbon Xerogels as Reusable Catalysts for Cyclohexane C-H Activation in the Presence of CO and Water

**DOI:** 10.3390/molecules22040603

**Published:** 2017-04-09

**Authors:** Ana Paula da Costa Ribeiro, Luísa Margarida Dias Ribeiro de Sousa Martins, Sónia Alexandra Correia Carabineiro, José Luís Figueiredo, Armando José Latourrette Pombeiro

**Affiliations:** 1 Centro de Química Estrutural, Instituto Superior Técnico, Universidade de Lisboa, Av. Rovisco Pais, 1049-001 Lisboa, Portugal; apribeiro@tecnico.ulisboa.pt (A.P.C.R.); pombeiro@tecnico.ulisboa.pt (A.J.L.P.); 2 Chemical Engineering Department, Instituto Superior de Engenharia de Lisboa, Instituto Politécnico de Lisboa, R. Conselheiro Emídio Navarro, 1959-007 Lisboa, Portugal; 3 Laboratório de Catálise e Materiais, Laboratório Associado LSRE-LCM, Faculdade de Engenharia, Universidade do Porto, Rua Dr. Roberto Frias, 4200-465 Porto, Portugal; jlfig@fe.up.pt

**Keywords:** gold nanoparticles, C-H activation, hydrocarboxylation, cyclohexane, catalyst recycling, water, green metric

## Abstract

The use of gold as a promotor of alkane hydrocarboxylation is reported for the first time. Cyclohexane hydrocarboxylation to cyclohexanecarboxylic acid (up to 55% yield) with CO, water, and peroxodisulfate in a water/acetonitrile medium at circa 50 °C has been achieved in the presence of gold nanoparticles deposited by a colloidal method on a carbon xerogel in its original form (CX), after oxidation with HNO_3_ (-ox), or after oxidation with HNO_3_ and subsequent treatment with NaOH (-ox-Na). Au/CX-ox-Na behaves as re-usable catalyst maintaining its initial activity and selectivity for at least seven consecutive cycles. Green metric values of atom economy or carbon efficiency also attest to the improvement brought by this novel catalytic system to the hydrocarboxylation of cyclohexane.

## 1. Introduction

The single-pot carboxylation of C_n_ alkanes to C_n+1_ carboxylic acids by CO is a particularly attractive alkane functionalization procedure [1,2,3,4,5,6,7,8,9,10,11,12,13,14,15,16,17], in view of the increasing industrial demand for carboxylic acids and of the drawbacks of their current synthetic methods [18,19,20,21]. However, catalytic carboxylation of saturated hydrocarbons, such as alkanes, requiring C-H activation, is a considerable chemical challenge, in particular for the least reactive lower alkanes (1 to 6 carbon atoms).

Fujuwara et al. [1,2,4] found that a cyclohexane undergoes carboxylation to cyclohexanecarboxylic acid (4.3% yield relative to the substrate) with CO and peroxodisulfate in trifluoroacetic acid (TFA) at 80 °C, catalysed by a Pd(II)/Cu(II) system. The strongly acidic medium is required due to the inertness of the alkane. 

In recent years, intensive research has been focused on the improvement of alkane carboxylation towards future sustainable carboxylic acid production [10,11,15,17,22], namely regarding the use of greener and safer solvents. Hydrocarboxylation of cyclohexane to cyclohexanecarboxylic acid with CO and water (72% yield), in the presence of peroxodisulfate oxidant, in water/acetonitrile medium at circa 50 °C and in the presence of a tetracopper(II) catalyst has been achieved [10]. In this improved system, water plays the roles of both reactant and solvent [10]. In contrast to the carboxylation in TFA [4,6,7,8], the carboxylation of cyclohexane by CO in the H_2_O/MeCN/K_2_S_2_O_8_ system proceeds to some extent in the absence of any metal catalyst, leading to the formation (up to 12% yield) of cyclohexanecarboxylic acid. However, it can proceed more efficiently in the presence of a metal (V, Mn, Fe or Cu) promotor [10,11,22], leading to higher yields of carboxylic acid.

In spite of the above achievements, so far any tested homogeneous catalytic systems [22] have the drawback of not being re-usable, thus the search for a more efficient and eco-friendly heterogeneous processes for the synthesis of such industrially important commodities continues. Gold catalysts are currently a “hot topic” of research, as they show application in many reactions of industrial and environmental importance [23,24,25,26,27,28]. Several variables have been considered as important factors influencing the structure, reactivity, and catalytic activity. Among them are the method of preparation, the nature of the support, and particularly, the gold nanoparticle size [23,24,25,26,27,28].

**Scheme 1:**
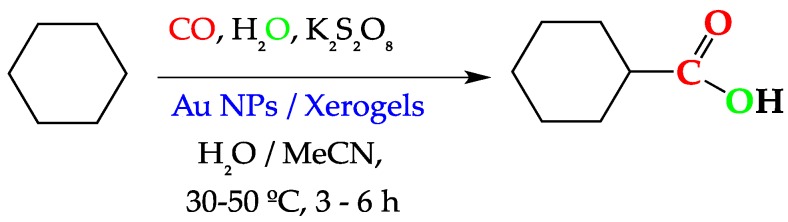
Hydrocarboxylation of cyclohexane to cyclohexanecarboxylic acid catalysed by gold nanoparticles supported on carbon xerogels.

Herein, we report the use of gold nanoparticles as promotors of cyclohexane hydrocarboxylation. We have chosen the above-mentioned protocol [10] and the use of gold as a metal promotor in view of the ability of [*^n^*Bu_4_N][AuCl_4_], Au C-scorpionate gold complexes, and Au nanoparticles to catalyze the peroxidative oxidation of cyclohexane to KA oil (cyclohexanol and cyclohexanone mixture) [29,30]. Moreover, gold nanoparticles are supported on carbon xerogels with different treatments, in order to provide recyclable catalysts for the one-pot hydrocarboxylation of cyclohexane to cyclohexanecarboxylic acid (Scheme 1).

To the best of our knowledge, this is the first report dealing with hydrocarboxylation of alkanes using gold nanoparticles as catalysts. In fact, the only reports found in literature so far, dealing with hydrocarboxylation of hydrocarbons using gold catalysts, refer to hydrocarboxylation of alkynes and to gold complexes (not gold nanoparticles) [31,32]. Moreover, the only report for hydrocarboxylation using carbon materials deals with 1,3-butadiene using a Rh(I) complex immobilized on activated carbon as the catalyst [33]. Therefore, our work is also the first report of such reaction carried out using carbon xerogel based catalysts.

## 2. Results and Discussion

### 2.1. Characterisation of Xerogel Supports

**Figure 1:**
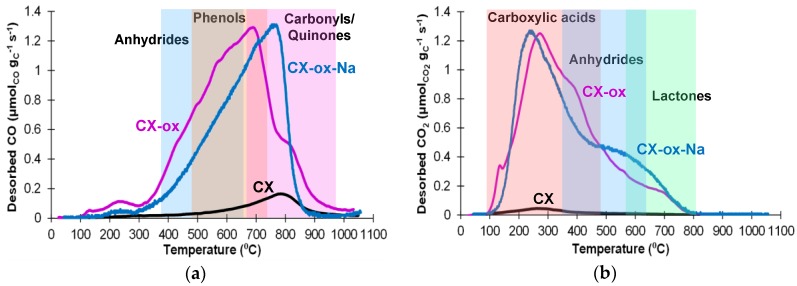
Temperature programed desorption (TPD) profiles for the carbon xerogel materials. Desorption of CO (**a**) and CO_2_ (**b**) is shown, with identification of types of groups desorbing in different temperature ranges (the different colour bars are only indicative of the temperature ranges expected for the desorption of different groups, and do not provide any information on their amounts).

The carbon xerogel was used as a support in its original form as prepared (CX), oxidised (-ox), and oxidised with nitric acid and subsequently treated with sodium hydroxide (-ox-Na). The characterization details of these samples can be found in Table 1 and Figure 1, which include the textural and surface characterisation. 

Table 1 shows that CX is mainly mesoporous and has a large pore size, as expected [29,30,34,35,36,37]. By comparing the parameters of the oxidized (CX-ox) samples with those of the parent material (CX), it is observed that liquid phase activation slightly decreased the surface area and pore volume, probably due to some pore wall collapse or to the presence of numerous oxygen-containing surface groups, which might partially block the access of N_2_ molecules to the smaller pores [37]. Nitric acid consumes large amounts of carbon atoms and changes the structure of pores, merging some of them together.

Figure 1 shows the identification of types of groups desorbing in different temperature ranges, according to what is already established in the literature [37,38,39,40,41]. Upon oxidation treatment, the amounts of CO and CO_2_ increase enormously (Table 1 and Figure 1). Figure 1a shows the CO desorption profiles. The effect of oxidation treatments is also seen in these data. The largest CO evolution of the -ox materials starts at around 350 °C, whereas for the -ox-Na samples, the temperature is slightly higher. That can be due to the destruction of carboxylic anhydrides (that desorb as CO and CO_2_ in that temperature range [37,38,39,40,41]) also contributing to the increase of the carboxylate groups, as proposed in literature [42]. Moreover, the profile of -ox-Na is a little sharper, more intense, and has its maximum at a higher temperature than that of the -ox sample. This suggests that phenol groups (that desorb as CO [37,38,39,40,41]) are converted into phenolates, which are more stable [42]. The CO_2_ desorption profiles (Figure 1b) of the -ox and -ox-Na materials also show a considerable increase in the amount of carboxylic acid groups (which decompose in the temperature range 200–350 °C, as described in the literature [37,38,39,40,41]) when compared to the original materials.

### 2.2. Characterisation of Gold Catalysts

The nominal gold loading was 3% wt. (see Materials and Methods). However, as shown in Table 2, only CX showed an actual loading near that value (2.8%). Regarding the functionalized samples, it has been reported that surface oxygen groups can act as anchoring sites for metallic precursors [43,44]. However, in the particular case of Au loaded on xerogels by the colloidal method used in this work (see Materials and Methods), it seems that the presence of oxygenated groups is detrimental for Au loading, as much less gold was loaded on CX-ox (1.4%) and even less on CX-ox-Na (0.5%), as depicted in Table 2.

**Figure 2:**
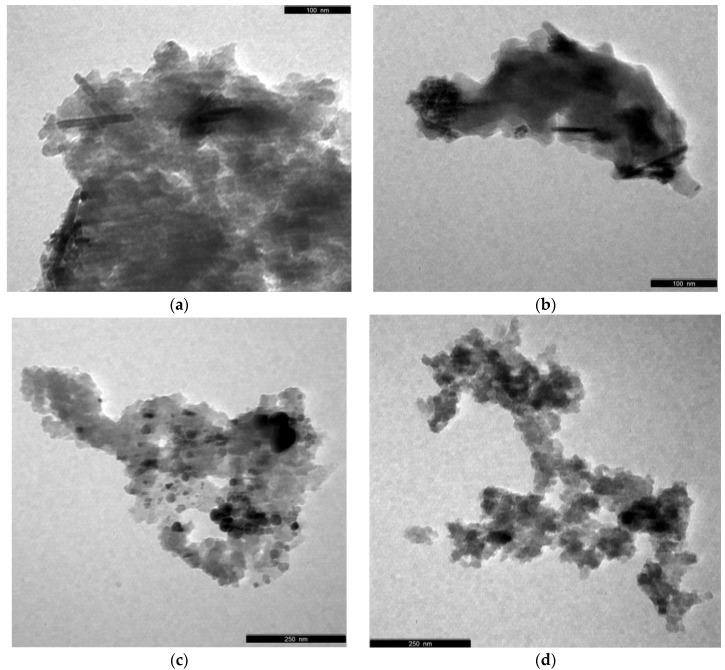
TEM images carbon xerogel samples: CX (**a**,**b**), CX-ox (**c**) and CX-ox-Na (**d**). Gold nanoparticles are seen as darker spots, with rod-like (**a**,**b**), or spherical/obliquous (**c**,**d**) shapes.

Figure 2 shows some selected transmission electron microscopy (TEM) images of the samples. It can be seen that gold nanoparticles are deposited mostly in the form of nanorods on CX (Figure 2a,b). Although spherical nanoparticles are usually obtained with the colloidal method [30,45,46,47], nanorods are also often reported in literature [48,49]. They are usually formed through a seed-mediated method, which includes the formation of “seed” nanoparticles and the growth of such seeds into rods [49]. Also other agglomerates of particles are seen (Figure 2b). Au on CX-ox and CX-ox-Na (Figure 2c,d, respectively) show more regular sphere-like particles, which are larger on CX-ox (Figure 2c).

Table 2 shows a summary of the values of the average gold nanoparticle size and dispersion. It can be seen that the average size is larger for CX (16.6 nm, calculated only for spherical nanoparticles, as nanorods larger than 100 nm are also observed—Figure 2a,b). 14.2 nm was found for CX-ox and 13.7 nm for CX-ox-Na. In both cases, there was agglomeration of gold nanoparticles (one example is shown in Figure 2c). Consequently, dispersion is smaller on CX and larger on CX-ox-Na, although the value of 8.4% can still be considered low.

In a previous work of ours, dealing with gold nanoparticles on several carbon materials, including xerogels [30], for 1% Au loading, spherical nanoparticles of ca. 4.4 nm were obtained with a metal dispersion of 26.2%. Most likely, the larger loading used in this work promoted agglomeration as well as nanorods formation in the case of CX. Although apparently detrimental for gold loading, the presence of surface oxygenated groups seems beneficial for the formation of spherical, less agglomerated nanoparticles. As stated above, it was previously reported that surface oxygen groups can act as anchors for the metallic precursors [43,44] and that can result in smaller and better dispersed nanoparticles (at least compared with the unfunctionalized support).

### 2.3. Catalytic Results

**Figure 3:**
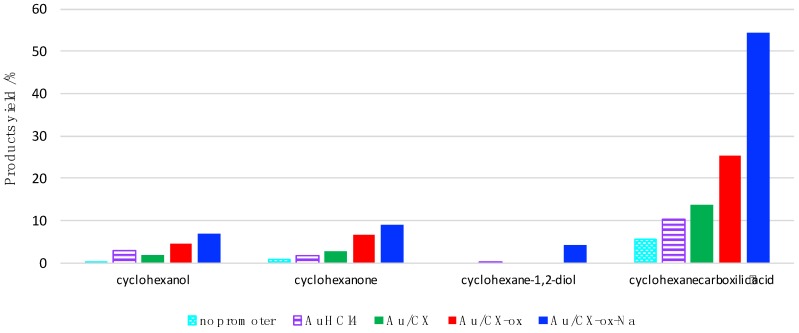
Products yields from cyclohexane hydrocarboxylation: metal-free (■), promoted by HAuCl_4_.3H_2_O in homogeneous conditions (■) and by Au NPs deposited on different carbon xerogels: as prepared (CX) (■), treated with nitric acid (CX-ox) (■) and oxidized with nitric acid and subsequently treated with sodium hydroxide (CX-ox-Na) (■).

Gold nanoparticles supported on different carbon xerogel samples exhibited different catalytic activities (Figure 3). The desired cyclohexanecarboxylic acid was achieved with up to 54.5% yield with Au on CX-ox-Na (entry 1, Table 3). However, KA oil (cyclohexanol and cyclohexanone mixture) and cyclohexane-1,2-diol were also obtained, although in much lower (<10%) yields. The conversion of cyclohexane to cyclohexanecarboxylic acid (and also to the other oxidation products) follows the order CX-ox-Na > CX-ox > CX (Figure 3). This can be related with the smaller gold nanoparticle size found on CX-ox-Na and CX-ox, compared to that of CX (Table 2), which is expected to affect catalytic activity [23,24,25,26,27,28].

It is also well known that the presence of alkali metals enhances the activity of gold catalysts [50,51,52]. Thus, the presence of sodium carboxylate and phenolate groups might also be beneficial to the catalytic activity (although the supports alone, without gold, revealed no catalytic activity). Higher activities (for the same Au amount and reaction conditions) were found for gold nanoparticles deposited on carbon xerogel samples, when compared to HAuCl_4_·3H_2_O used in homogeneous medium, i.e., in aqueous solution (Figure 3, Table 3).

The catalytic activity of gold nanoparticles on xerogels is also dependent on the reaction conditions. It was found that high pressures of CO do not enhance the production of carboxylic acid, the best being the 1:1 molar ratio of CO relative to cyclohexane (compare entries 1 and 2 of Table 3). In all hydrocarboxylation systems known to date, a 10:1 molar excess of CO relative to substrate is required (see conditions of Table 4) [22]. This is a very important advantage for our system, in terms of the environment and in process safety.

Moreover, the Au/CX-ox-Na/CO/K_2_S_2_O_7_/H_2_O/MeCN system exhibits its maximum performance at the mild temperature of 50 °C (compare entries 1, 3 and 4, Table 3) requiring a significantly (up to 16 times) lower amount of metal promoter than in the previously reported systems (Table 4). In fact, considering the cyclohexanecarboxylic acid yield per amount of metal promotor (Table 4) the Au/CX-ox-Na system exhibits significantly better performance on cyclohexane hydrocarboxylation relative to the formerly tested promotors [10] (cyclohexanecarboxylic acid yield is 1.5 times higher than that of the literature best catalyst [OCu_4_{N(CH_2_CH_2_O)_3_}_4_(BOH)_4_][BF_4_]_2_, entries 4 and 16, respectively, of Table 4).

In addition, the present catalytic system was evaluated by green metrics such as atom economy (molecular weight of desired product per combined molecular weight of starting materials) or carbon efficiency (amount of carbon in CyCOOH per total carbon in reactants). Such metrics were not included in the previously reported systems. Thus, to compare our system with the previous ones (reported in [10]), the carbon efficiency and the atom economy were determined (Table 4). Although presenting the same atom economy value, our gold systems exhibit markedly higher carbon efficiency values (Table 4), which is a significant improvement in terms of the sustainability of the hydrocarboxylation reaction.

**Figure 4:**
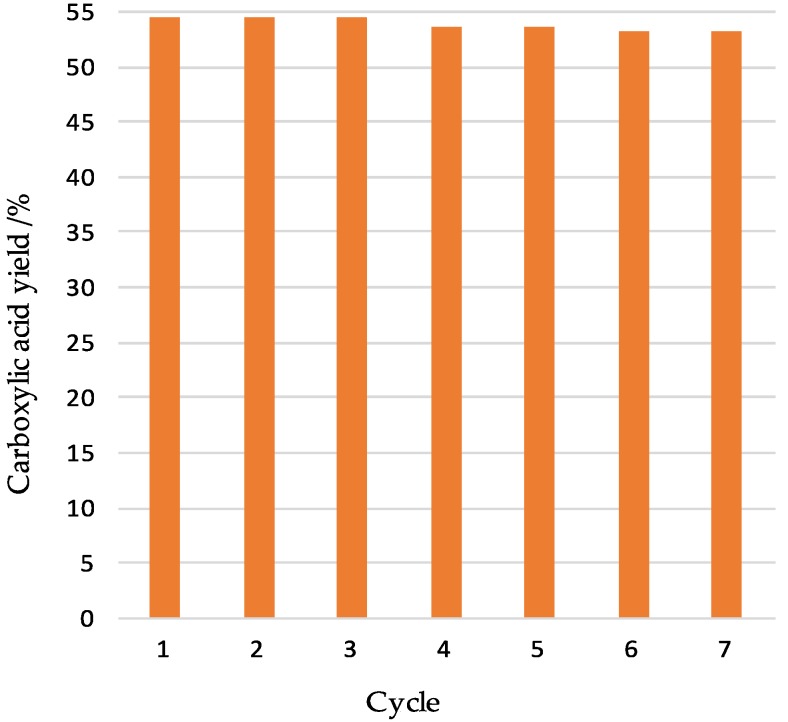
Effect of the catalyst recycling on the yield of cyclohexanecarboxylic acid obtained by hydrocarboxylation of cyclohexane catalyzed by Au/CX-ox-Na. Reaction conditions: cyclohexane (1.00 mmol), p(CO)= 2 atm, K_2_S_2_O_8_ (1.50 mmol), catalyst (2 μmol), H_2_O (3.0 mL)/MeCN (3.0 mL), 50 °C, and six hours in an autoclave (13.0 mL capacity).

Another important advantage of the present systems is the possibility of being recycled and re-used. Recycling of the best catalyst, Au/CX-ox-Na, was tested on up to seven consecutive cycles. On completion of each stage, the products were analyzed and the catalyst was recovered by filtration, thoroughly washed, and then reused for a new set of cyclohexane hydrocarboxylation experiments. The filtrate was tested in a new reaction (by addition of fresh reagents), and no oxidation was detected. Figure 4 shows the excellent recyclability of the system Au/CX-ox-Na: in the second, third, fourth, fifth, sixth, and seventh run, the observed activity was 99.8%, 99.7%, 98.4%, 98.3%, 97.7% and 97.5% of the initial one, being the selectivity maintained.

## 3. Materials and Methods

### 3.1. Reagents

All the reagents and solvents were purchased from commercial sources and used as received. The water used for all reactions and analyses was double distilled and deionised.

### 3.2. Carbon Materials Preparation

Carbon xerogel (CX) was prepared by polycondensation of resorcinol and formaldehyde, using a pH of 6, according to a procedure described elsewhere [29,30,34,35,36,37]. It was used in its original form (CX), oxidized (-ox), and oxidized with nitric acid and subsequently treated with sodium hydroxide (-ox-Na). CX-ox was obtained by refluxing CX with 75 mL of a 5 M nitric acid solution, per gram of carbon material, for 3 h, then separated by filtration and washed with deionized water until neutral pH, similarly to what was reported earlier [29,35,36,37]. CX-ox-Na was obtained by treating CX-ox with 75 mL of a 20 mM NaOH aqueous solution, per gram of carbon material, in reflux for 1 h, as reported in the literature [29,35,36]. This material was also separated by filtration and washed until neutral pH. 

### 3.3. Carbon Materials Characterisation

The carbon materials were characterised by N_2_ adsorption at 77 K in a Quantachrome Nova 4200e apparatus (Boynton Beach, FL, USA), using the Brunauer-Emmett-Teller (BET) theory for total surface area determination, Barrett-Joyner-Halenda (BJH) for pore size distribution and Boer’s t-method for micropore volume and external surface area. Their surface chemistry was characterised by temperature programed desorption (TPD) using an Altamira AMI-300 apparatus (Pittsburgh, PA, USA), with a coupled Ametek Dycor DyMaxion quadrupole mass spectrometer (Pittsburgh, PA, USA).

### 3.4. Gold Loading

Gold (nominal 3% wt) was loaded on the xerogel supports by the colloidal method [30,45,46,47], which consists of dissolving the gold precursor, HAuCl_4_·3H_2_O (Alfa Aesar, Karlsruhe, Germany), in water, adding polyvinyl alcohol (Aldrich, Darmstadt, Germany) and NaBH_4_ (Aldrich), resulting in a ruby red solution to which the xerogel support was added under stirring. After a few days, the solution of CX started to lose colour, as Au was deposited on the support. The colourless solution was filtered, the catalyst washed thoroughly with distilled water until the filtrate was free of chloride and dried at 110 °C overnight. Solutions of CX-ox and CX-ox-Na took more time and even so the deposition was not complete (as found out later when the amount of gold present was determined), as these solutions never turned colourless. However, the same filtering and washing procedures were followed as with CX. The organic scaffold was removed from the supports by heat treatment under N_2_ flow for 3 h at 350 °C (shown by elemental analysis to be efficient for this purpose), and then, the catalyst was activated by further treatment under hydrogen flow for 3 h also at 350 °C.

### 3.5. Gold Catalysts Characterisation

The Au/xerogel samples were imaged by transmission electron microscopy (TEM). The analyses were performed on a Leo 906E apparatus (Austin, TX, USA), at 120 kV. Samples were prepared by ultrasonic dispersion in hexane and a 400 mesh formvar/carbon copper grid (Agar Scientific, Essex, UK) was dipped into the solution for TEM analysis.

The average gold particle size was determined from measurements made on about 300 particles. The metal dispersion was calculated by D_M_ = (6n_s_M)/(ρNd_p_), where n_s_ is the number of atoms at the surface per unit area (1.15 × 10^19^ m^−2^ for Au), M is the molar mass of gold (196.97 g mol^−1^), ρ is the density of gold (19.5 g cm^−3^), N is Avogadro’s number (6.023 × 10^23^ mol^−1^) and d_p_ is the average particle size (determined by TEM, assuming that particles are spherical).

In order to determine the loading of gold, samples were incinerated at 600 °C and the resulting ashes were dissolved in a concentrated HNO_3_ and H_2_SO_4_ mixture. The resulting solution was diluted and analysed by atomic absorption spectroscopy (AAS) using a Unicam 939 atomic absorption spectrometer (Kent, UK) and a hollow cathode lamp Heraeus 3UNX Au.

### 3.6. Catalytic Tests

The single-pot reactions were carried out in stainless steel autoclaves, by reacting, at typical temperatures of 30–80 °C and in a water/acetonitrile medium with cyclohexane, carbon monoxide (pressures from 2 to 20 atm), gold catalyst (2–20 μmol) and potassium peroxodisulfate. The reaction mixture was stirred for 3–6 h (typically 6 h) using a magnetic stirrer and an oil bath, whereupon it was cooled in an ice bath, degassed, opened, and the contents transferred to a Schlenk flask. Diethyl ether (9.0–11.0 mL) and 90 mL of cycloheptanone (GC internal standard) were added. The obtained mixture was vigorously stirred for 10 min, and the organic layer was analysed typically by gas chromatograph (GC). A Fisons Instruments GC 8000 series gas chromatograph (Agilent Technologies, Santa Clara, CA, USA) with a DB-624 (J&W) capillary column (flame ionization detector) and the Jasco-Borwin v.1.50 software (Jasco, Tokyo, Japan) were used. GC-MS analyses were performed in a Perkin Elmer Clarus 600 C GC-MS instrument (Shelton, Connecticut, USA) equipped with a 30 m × 0.22 mm × 25 μm BPX5 (SGE) capillary column, using He as the carrier gas. The internal standard method was used to quantify the organic products, since the desired cyclohexanecarboxylic acid was not isolated from the reaction mixture.

Blank tests (i) without any catalyst; (ii) only with CX, CX-ox and CX-ox-Na; and (iii) using only one of the solvents (H_2_O or NCMe) were also performed, to assess if the carboxylation reactions proceeded in the absence of the metal catalyst, and the importance of each support and solvent. Moreover, aqueous solutions of the gold precursor were also tested for comparison (homogenous medium).

## 5. Conclusions

Gold nanoparticles were successfully deposited on carbon xerogel, as prepared and with different treatments: with nitric acid; and oxidized with nitric acid and subsequently treated with sodium hydroxide. The catalytic activity of the said materials was assessed for the single-pot hydrocarboxylation of cyclohexane, in H_2_O/MeCN, under mild conditions (50 °C, 2 atm of CO). Au/CX-ox-Na exhibited the best performance, yielding cyclohexanecarboxylic acid up to 54.5% yield, and excellent recyclability, maintaining 97.5% of the initial activity after seven consecutive catalytic cycles. Green metric values of carbon efficiency also confirmed the improvement brought by this novel catalytic system to the hydrocarboxylation of cyclohexane.

These results have an important implication on the design of gold catalysts and are of potential significance for the sustainable production of carboxylic acids.

## Figures and Tables

**Table 1:** Description and characterisation of carbon xerogel samples: surface area (S_BET_), total pore volume (V_p_), average mesopore width (L), micropore volume (V_micro_), external area (S_external_), obtained by adsorption of N_2_ at −196 °C, and amounts of CO and CO_2_ desorbed, as determined by temperature programmed desorption (TPD).

Sample	S_BET_ (m^2^/g)	V_p_ (cm^3^/g)	L (nm)	V_micro_ (cm^3^/g)	S_external_ (m^2^/g)	CO (µmol/g)	CO_2_ (µmol/g)
CX	604	0.91	13.7	~0	604	492	135
CX-ox	570	0.80	18.8	0.038	512	4609	3774
CX-ox-Na	560	0.75	17.6	0.036	496	3720	3793

**Table 2:** Average gold nanoparticle size and dispersion (calculated from TEM measurements) and gold loading (calculated by atomic absorption spectroscopy) on the different carbon xerogel materials.

Sample	Au Average Size (nm)	Metal Dispersion (%)	Gold Loading (%)
Au/CX	16.6 *	6.9 *	2.8
Au/CX-ox	14.2	8.1	1.4
Au/CX-ox-Na	13.7	8.4	0.5

*—calculated taking into account only the spherical nanoparticles, not the nanorods.

**Table 3:** Selected data *^a^* for cyclohexane hydrocarboxylation promoted by Au/CX-ox-Na.

Entry	Au/μmol	P(CO)/atm	Temperature/°C	Total TON *^b^*	Yield/% *^c^* CyCOOH Cy_-H_=O CyOH Cy_-H_(OH)_2_			
1	2	2	50	375	54.5	9.1	7.1	4.3
2	2	20	50	311	28.3	19.4	14.5	3.9
3	2	2	30	111	12.0	5.3	4.9	0.8
4	2	2	80	245	24.1	14.4	9.5	0.9
5	20	2	50	23	19.9	12.3	12.5	0.5

*^a^* Reaction conditions: cyclohexane (1.00 mmol), p(CO) = 2–20 atm, K_2_S_2_O_8_ (1.50 mmol), catalyst (2–20 μmol), H_2_O (3.0 mL)/MeCN (3.0 mL), 30–80 °C, 6 h in an autoclave (13.0 mL capacity); *^b^* Turnover number = moles of products per mole of catalyst; *^c^* Moles of product per 100 mol of cyclohexane; Cy = C_6_H_11_.

**Table 4:** Metal promotors performance comparison *^a^* for the cyclohexane hydrocarboxylation to cyclohexanecarboxylic acid.

Entry	Metal Promoter	CyCOOH Yield (%) *^b^*/μmol of M Promotor	Carbon Efficiency/% *^c^*	Atom Economy/% *^d^*
1	HAuCl_4_·3H_2_O *^e^*	5.2	7.7	33.5
2	Au/CX *^e^*	6.9	9.3	
3	Au/CX-ox *^e^*	12.7	18.4	
4	Au/CX-ox-Na *^e^*	27.3	37.9	
5	Cr(OH)_3_·2.5H_2_O [10]	0.4	0.6	33.5
6	K_2_Cr_2_O_7_ [10]	1.0	3.9	
7	MoO_3_ [10]	0.0	0.0	
8	H_4_[PMo_11_VO_40_]·34H_2_O [10]	0.4	1.1	
9	MnO_2_ [10]	0.5	1.4	
10	Fe(OH)_3_·0.5H_2_O [10]	1.0	1.6	
11	Co(acac)_3_ [10]	0.6	0.9	
12	Zn(NO_3_)_2_ [10]	0.5	0.8	
13	Cu(NO_3_)_2_·2.5H_2_O [10]	1.0	3.3	
14	[Cu(H_2_tea)(N_3_)] [10]	2.0	3.3	
15	[Cu_2_(H_2_tea)_2_(tpa)]_n_·2nH_2_O [10]	4.9	3.8	
16	[OCu_4_{N(CH_2_CH_2_O)_3_}_4_(BOH)_4_][BF_4_]_2_ [10]	18.1	7.1	

*^a^* Typical (unless otherwise stated) reaction conditions: cyclohexane (1.00 mmol), p(CO) = 20 atm, K_2_S_2_O_8_ (1.50 mmol), H_2_O (3.0 mL)/MeCN (3.0 mL), 50 °C, 6 h in an autoclave (13.0 mL capacity); *^b^* Moles of cyclohexanecarboxylic acid per 100 mol of cyclohexane; Cy = C_6_H_11_; ***^c^*** Amount of carbon in CyCOOH *per* total carbon in reactants × 100%; *^d^* Molecular weight of desired product per combined molecular weight of starting materials × 100%; *^e^* p(CO) = 2 atm.
